# The pristine precursor of Andean-type magmatism preserved in magma mingling zones

**DOI:** 10.1038/s41598-024-55699-x

**Published:** 2024-02-29

**Authors:** Daniel Gómez-Frutos, Antonio Castro, Jesús de la Rosa

**Affiliations:** 1 Museo Nacional de Ciencias Naturales (MNCN), Consejo Superior de Investigaciones Científicas (CSIC), C. José Gutiérrez Abascal 2, 28006 Madrid, Spain; 2 Centro de Investigación CIQSO, Universidad de Huelva, Huelva, Spain

**Keywords:** Geochemistry, Petrology

## Abstract

Intermediate magma compositions have been postulated to be parental to Andean-type magmatism in the recent years. Geochemical and experimental methods have allowed the modelling of a hypothetical parental composition that accounts for the major element trends displayed by Andean-type batholiths. However, natural plutonic examples matching the modelled composition remain lacking, likely due to the predominance of fractionated liquids and cumulates in the batholiths after protracted and large-scale differentiation. Contrary to this, magma mingling zones, a common feature in Andean-type batholiths, are characterised by quenching phenomena, minimising differentiation. In this paper, we present data from intermediate magmatism in the world-class Gerena magma mingling zone in the Seville Sierra Norte batholith (southern Iberia), compositionally equivalent to Andean-type magmatic series. Geochemical data from quenched dark globules of variable scale and the corresponding host granodiorites are contrasted with the bimodal trends displayed by the host batholith. Results suggest that the smaller-scale dark globules have not undergone any significant fractionation. Furthermore, after conducting geochemical modelling we conclude the dark globules represent a composition that could be parental to Andean-type magmas. We propose that magma mingling zones are an optimal place to probe for parental magmas of Andean-type magmatism, particularly those represented in pristine melanocratic, intermediate globules.

## Introduction

Intermediate composition volcanic rocks and magmas are volumetrically dominant over basalts in active continental margins^1^. Similarly, diorites and Qz-diorites, the plutonic equivalents of erupted andesites, dominate over gabbros. Intermediate magma compositions (SiO_2_ = 53–63 wt%; MgO = 2–8 wt%) have been extensively used as parental magma systems for experimental modelling of the compositional trends of batholiths^2–11^. However, unambiguous identification of intermediate plutonic rocks matching the model compositions remains elusive.

This problem relates to one of the main features displayed by Andean-type batholiths, namely the existence of compositional gaps^12,13^. Commonly known as the Bunsen-Daly gap^14,15^, these refer to two compositional groups (low silica and high silica) separated by a silica interval (around 55–60 wt% SiO_2_) with scarce representation from natural samples. Different plausible explanations include that compositional gaps result when a hot, mantle-sourced basalt intrudes the lower crust and partially melts it to produce a silicic magma^14,16,17^; large-scale magma immiscibility^18^; or continuous differentiation that gives rise to cumulates and fractionated liquids^19^. Whatever the case, these processes preclude the identification of rocks as parental magmas, highlighting the relevance of identifying zones where fractionation was limited or non-existent. Moreover, all these models entail the coexistence of different magma types in the same chamber, producing local yet pervasive mingling phenomena in the batholiths^20,21^.

In this sense, magma mingling zones (MMZ), a common feature of Andean-type batholiths, drew the attention of volcanologists because they represent snapshots of the complex dynamic processes that occurred in response to magma chamber replenishment^22^. Quenching phenomena, a characteristic feature of MMZ due to the thermal contrast between different types of magmas, minimise magmatic differentiation, mixing and contamination with the host crust. Thus, quenching maintains almost intact the pristine composition of the magmas invading shallow felsic magma chambers by replenishment. Building on this foundation, we conducted a geochemical study of mingled Andean-type intermediate magmas in the world-class example of Gerena MMZ (Seville Sierra Norte batholith, Southern Spain), where details of magma mingling were reported years ago^23^. A preliminary comparison between Gerena, the Variscan Seville Sierra Norte batholith and the Jurassic Guadalupe Igneous Complex (California) is used to establish the Andean-type affinity of Gerena MMZ. Subsequent geochemical criteria are then used for the identification of samples with limited fractionation. After geochemical modelling comprising existing data from the Seville Sierra Norte batholith, the results will provide insights into the identification of pristine, parental intermediate magmatism in Andean-type settings. Although this work is referred to plutonic systems, the geochemical equivalence between arc plutonic and volcanic rocks has been vastly addressed in the existing literature^24,25^, thus making the results of this work applicable to volcanic settings. We contend that the application of the same criteria to other MMZs around the world could be relevant in addressing petrogenetic studies of batholiths and silicic volcanism.

## Geological setting, field relations and sampling

**Figure 1:**
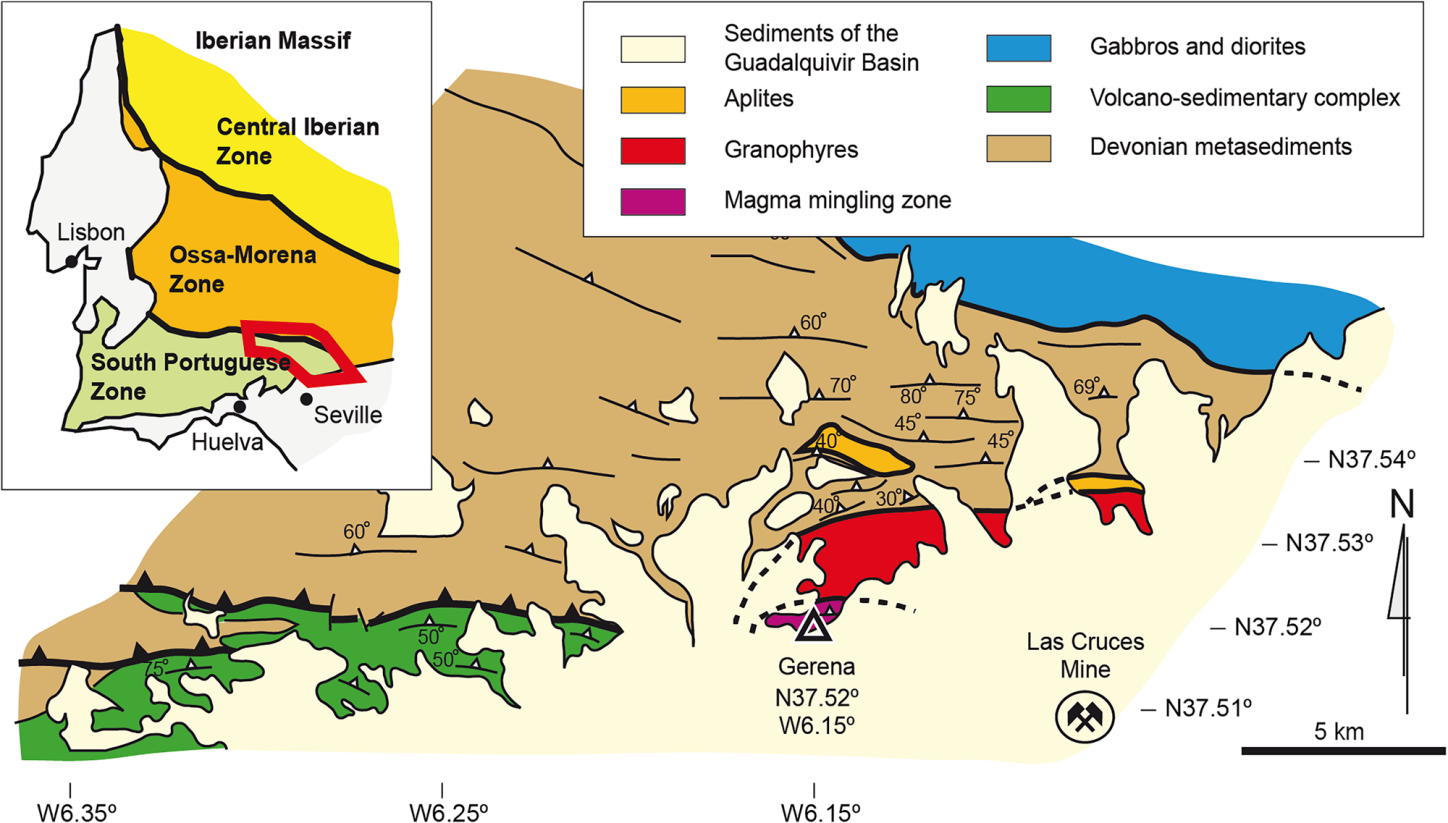
Geological map of the Seville Sierra Norte batholith showing the location of the Gerena magma mingling zone. Map modified from Instituto Geológico y Minero de España, mapa geológico digital continuo GEODE (accessed in 2023, available for public access in http://info.igme.es/cartografiadigital/geologica/geodezona.aspx?Id=Z1500). The map and the rest of the figures were created using Adobe Illustrator 27.9 under a license from the Museo Nacional de Ciencias Naturales (Consejo Superior de Investigaciones Científicas).

The Gerena MMZ is part of a major intrusive body of the Seville Sierra Norte batholith, emplaced during the Lower Carboniferous at the South Portuguese zone of the Iberian massif (Fig. 1). The Seville Sierra Norte batholith was emplaced at ca. 340 Ma^26^ into a low-grade metasedimentary pile formed by Devonian siliciclastic metasediments and metavolcanics and Lower Carboniferous volcano-clastic successions of the Iberian Pyrite belt. The whole batholith has been interpreted as Andean-type in terms of rock compositions, resulting by subduction and further oblique collision of the Laurussia and Gondwana supercontinents during the Carboniferous^27^. The Gerena MMZ is exceptionally exposed at the Guadalquivir fault, containing well preserved relations of magma-in-magma injections and quenching. Two main types of magmatism can be found, namely net-veined felsic complexes that intruded a consolidated tonalite, and intermediate globular intrusions and synplutonic dykes that intruded a felsic magma chamber^23^. A representative number of samples were collected from both the felsic complexes and the intermediate globules. However, since the felsic complexes expectedly represent differentiated magma, the present study mainly revolves around the melanocratic, intermediate globules.

**Figure 2:**
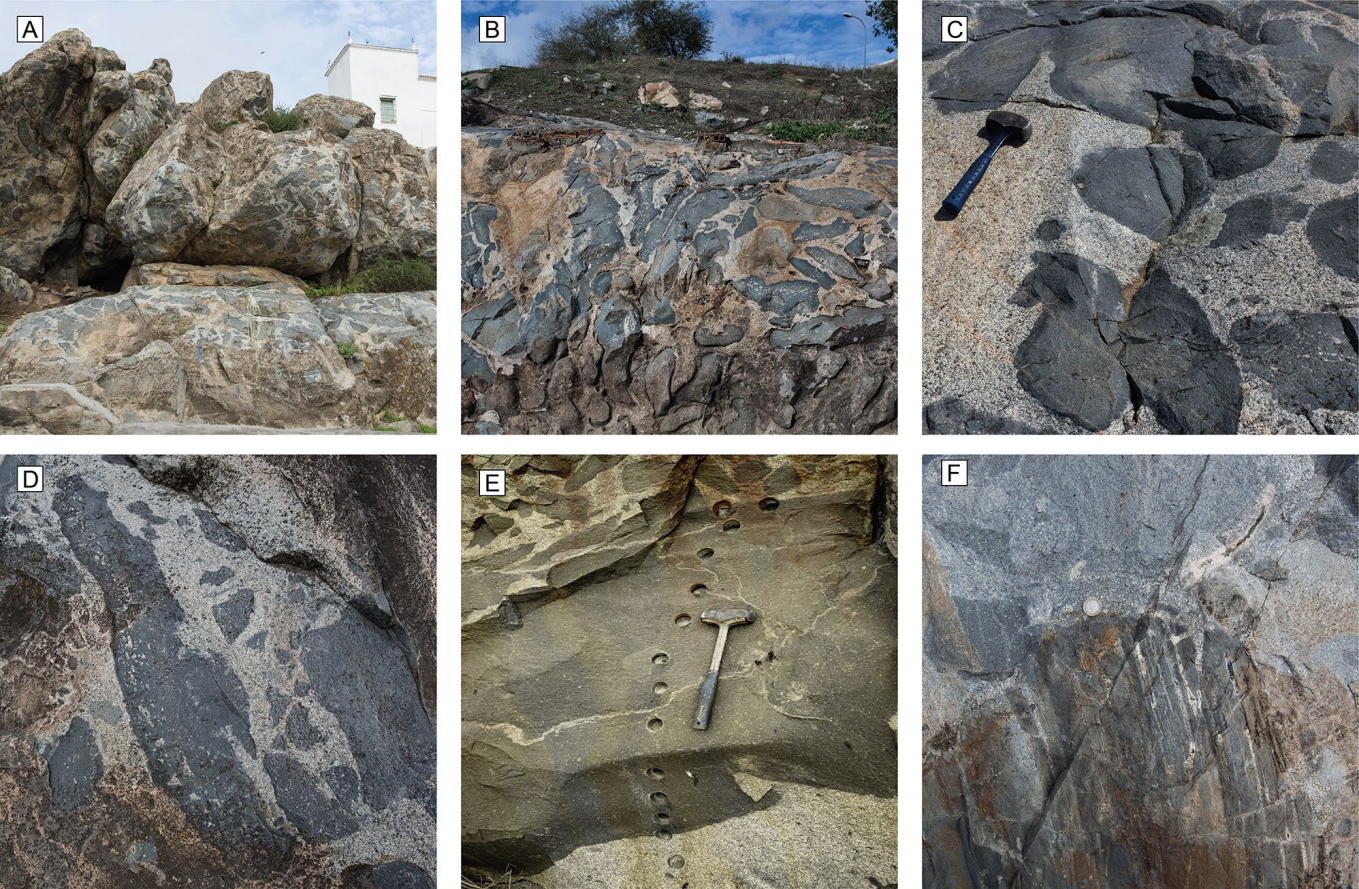
Representative field relations of dark globules and synplutonic dykes of the Gerena magma mingling zone. (**A, B**) General view of one of the breccia-like zones with dark magmatic globules of variable shape and size enclosed in a medium-grained biotite granodiorite. (**C**) Details of homogeneous dark globules with rounded shapes and devoid of continuous chilled margins. (**D**) Large and elongated globule showing pegmatitic vesicles concentrated along a zone close to the chilled margin on one side. The other side shows irregular contacts with dispersed fragments around. (**E**) Transversal section of a large dark globule from the chilled margin to the interior. Fragments from the chilled margin are observed as inclusions (autoliths) in the interior of the globule. (**F**) Partially molten hornfels xenolith from metasedimentary country rocks. These xenoliths supply evidence for local contamination of the host granodiorite.

Diorites and Qz-diorites manifest as distinct, dark fine-grained globules that exhibit diverse sizes and shapes, spanning from a few centimetres to several meters. These globules can display angular or rounded forms and are readily identifiable by their overall fine-grained textures, occasionally porphyritic, and non-crenulated contacts. They are found within a matrix of medium-grained biotite-granodiorite (Fig. 2a,b). Dark globules occasionally display discrete and discontinuous chilled margins with finer grain size than the core, and lobate contacts against the host granodiorite (Fig. 2c). Some dark globules contain pegmatitic ocelli aligned in bands parallel to the chilled margins (Fig. 2d) and represent fragments from the external rigid carapace of magma injections. Larger dark globules also found in the Gerena MMZ appear as meter-sized dismembered bodies, displaying a tabular geometry and chilled margins (Fig. 2e). Hornfels xenoliths from the host rocks are found scattered in the granodiorites, showing small degree of migmatization (Fig. 2f).

Short-range differentiation by in-situ crystallization, in conjunction with the formation of chilled margins in ascent conduits and dykes^28^, may imply small variations in silica ranges in larger dark globules. Thus, in order to identify undifferentiated magma compositions, we test the presence of minor variations in globules of variable scale. A set of samples was collected across a large dark globule showing a chilled margin at one of the sides (Fig. 2e). Other samples were collected from smaller dark globules. A few samples from the host granodiorites were also collected for reference. The dark globules appear as fine-grained, slightly porphyritic rocks constituted by plagioclase, amphibole and quartz as the main phases, and biotite, apatite, K-feldspar, titanite, zircon and opaque minerals as accessory phases. On the other hand, the host granodiorites are characterised by a medium-grain, locally granophyric texture, and are mainly composed of quartz, K-feldspar and plagioclase, and allanite, apatite, hornblende, and zircon as common accessory minerals. An exhaustive description of the Gerena rocks and mineral compositions can be found in previous works^23^.

Samples were crushed, milled, and analysed for major and trace elements using standard XRF and ICP-MS techniques, respectively. A selection of samples from dark globules were analysed for trace elements and Sr and Nd isotopes. The complete set of results can be found in the Supplementary Material S1, and the analytical details are given in the Supplementary Material S2.

## Whole rock geochemistry

**Figure 3:**
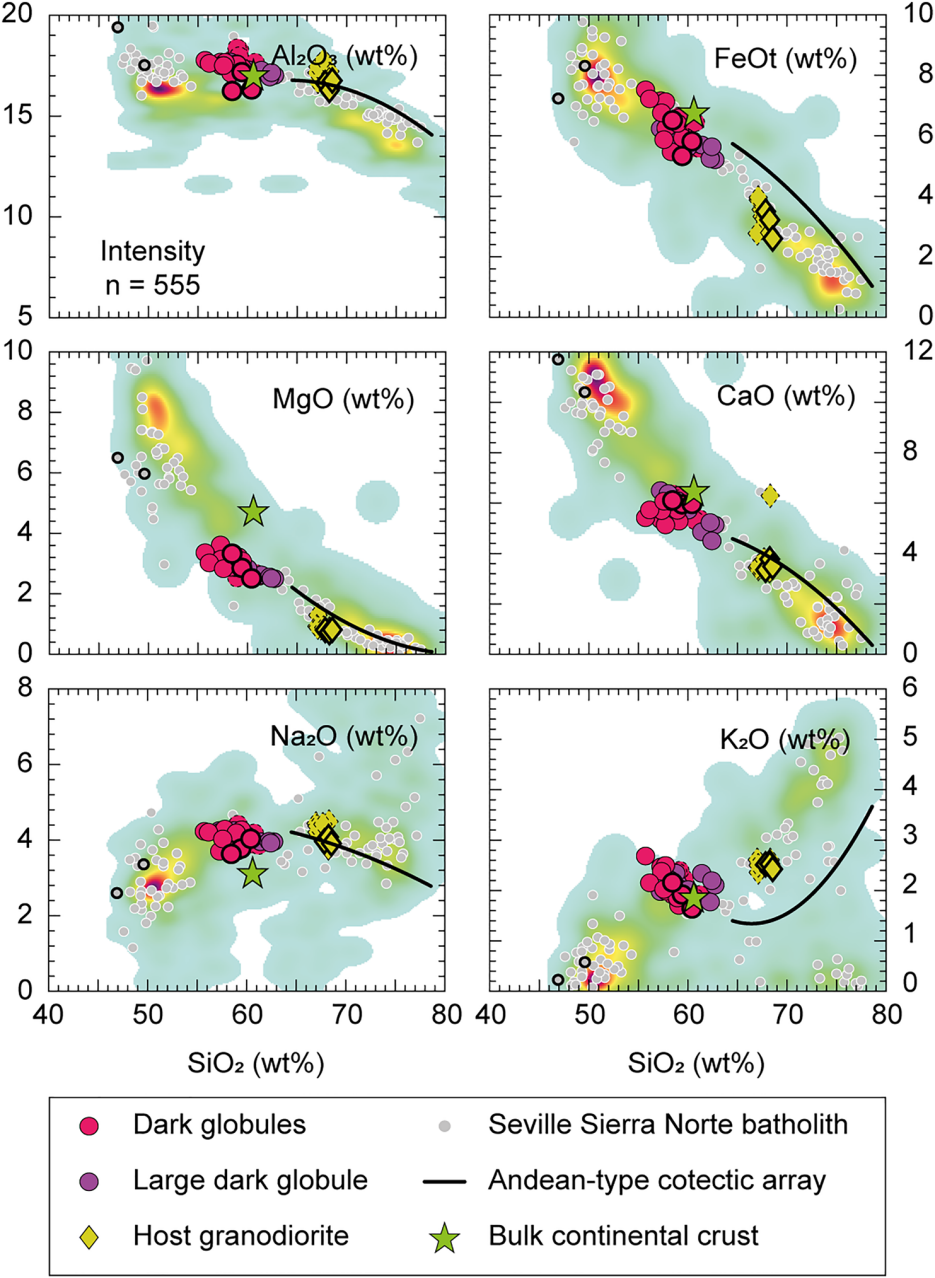
Harker diagrams for the Gerena samples. Comparative series from Sevilla Sierra Norte batholith^27^ and Guadalupe Igneous Complex^29^. Dashed points are additional Gerena analyses reported years ago^23^, from which the dark globules show an exact overlap with the new analyses. Points with thicker contours represent the compositions used for geochemical model in Fig. 6.

**Figure 4:**
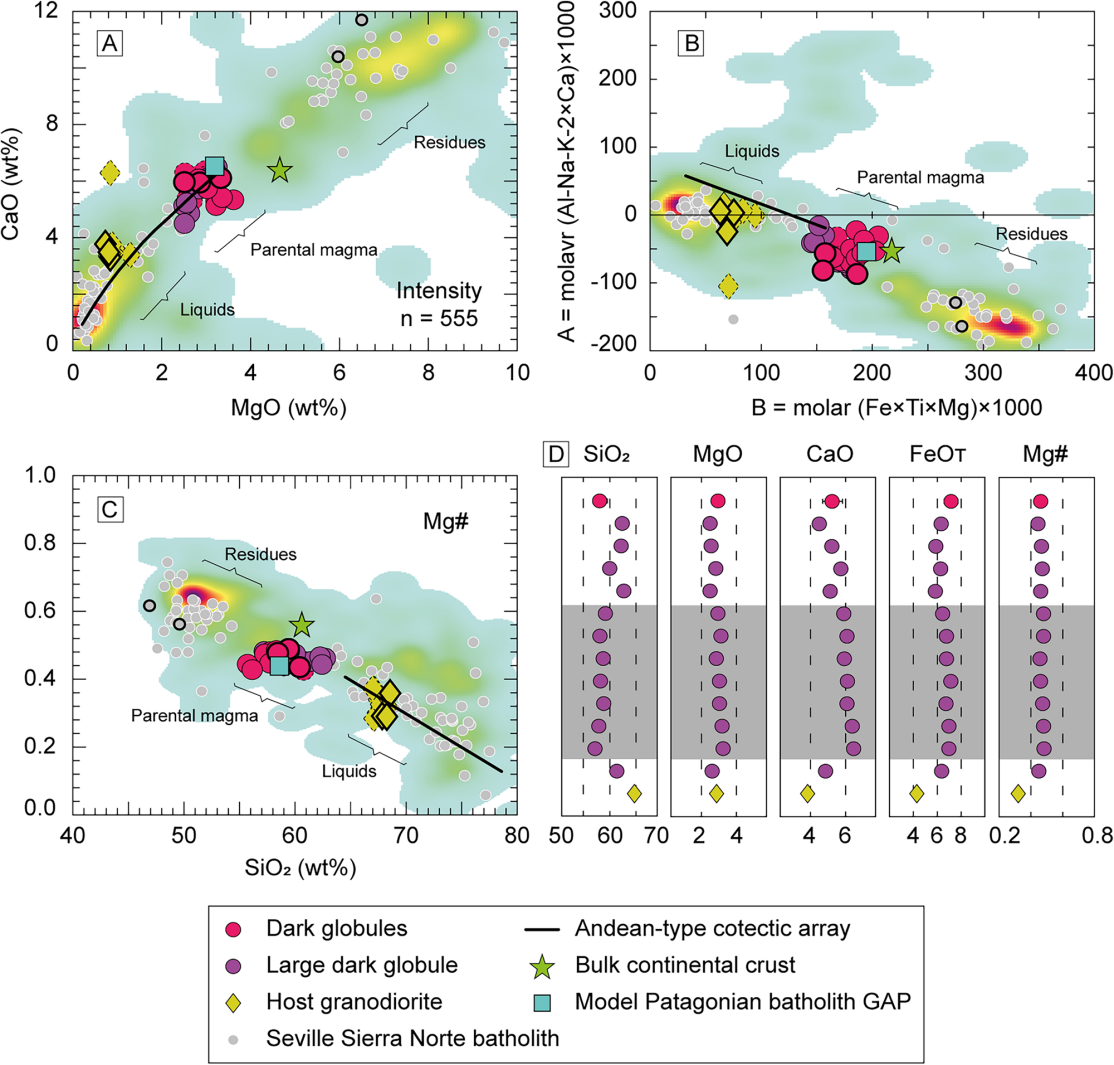
Classification diagrams for the Gerena samples and geochemical profile of the large dark globule. Reference GAP model is added for comparison^4^. Comparative series and colour codes are the same as in Fig. 3. (**A**) CaO–MgO diagram. All collected samples fall in the gap between liquids and residues. Most fractionated samples follow the trend displayed by the Andean main cotectic array (black line^4^). (**B**) A–B diagram. (**C**) Mg# against SiO_2_ diagram. (**D**) Compositional profile across the dyke shows the variations in major elements (numbers are wt%) resulting from differentiation by in-situ crystallization from the walls to inside. Shaded area represents samples matching the composition of the average of dark globules.

Comparative classification diagrams for Gerena MMZ samples, the Sevilla Sierra Norte batholith and experimental cotectic liquids are available in Figs. 3 and 4. Data from previous studies in the Gerena MMZ^23^ and the GAP model for Andean-type magmas parental^4^ were also plotted in the figures. A database from the Guadalupe Igneous Complex^29^ was represented for comparative purposes as a reference of a well-known Andean-type batholith (kernel density plot, Figs. 3, 4). Composition of the average bulk continental crust is available for further reference^30^. The exact overlap between the Sevilla Sierra Norte batholith and the Guadalupe Igneous Complex provides an immediate depiction of the Andean-type affinity of Gerena MMZ. Consistent with this pattern, all Gerena samples follow the trend displayed by the Guadalupe Igneous Complex in Harker diagrams, only diverging with slightly smaller CaO and MgO, and larger Al_2_O_3_ contents for their respective silica values (Fig. 3). Noticeable differences are observed between the dark globules and the larger dark globule, with the former clustering within a narrow silica interval (56–60 wt% SiO_2_) compared to the linear trend displayed by large dark globule. In the CaO–MgO (Fig. 4a), despite their lower CaO and MgO respective to silica contents, Gerena samples follow the experimental cotectic path of calc-alkaline rocks^4^, coherent with the patterns of Andean-type batholiths^24^. The A–B diagram (A = molar [Al–Na–K-2 × Ca] × 1000; B = molar [Fe + Mg + Ti] × 1000) presents a similar situation, although the large dark globule exhibit a scattered distribution instead of a linear pattern (Fig. 4b). Notably, the average bulk continental crust composition matches the dark globules from Gerena MMZ, with the only deviations found in MgO contents (Figs. 3, 4). Moreover, major element patterns in Figs. 3 and 4 showcase Gerena samples falling in the compositional gap of the Seville Sierra Norte batholith.

A geochemical profile of the large dark globule is shown in Fig. 4d, offering an enhanced visualization of the inner compositional zoning of the meter-sized dark globule. The average composition of the smaller dark globules is offered for comparison. Enrichment in SiO_2_ and depletions in FeO, MgO and CaO are observed in the edges of the profile. Contamination with the host granodiorite (yellow diamonds, Fig. 4d) is observed near the contact. However, composition remains unmodified over a wide zone of large globule (grey zone, Fig. 4d), most prominently showed by the constant Mg#, and close to the average composition of smaller dark globules (purple squares, Fig. 4d). Such geochemical homogeneity strongly suggests that there is no significant differentiation among the globules, with only small variations found in the rims. Plagioclase phenocrysts are mostly concentrated at the central part of the globule, giving rise to Ca and Al enrichment with respect to the chilled margins. Samples from the central zone overlap the composition of the smaller dark globules.

**Figure 5:**
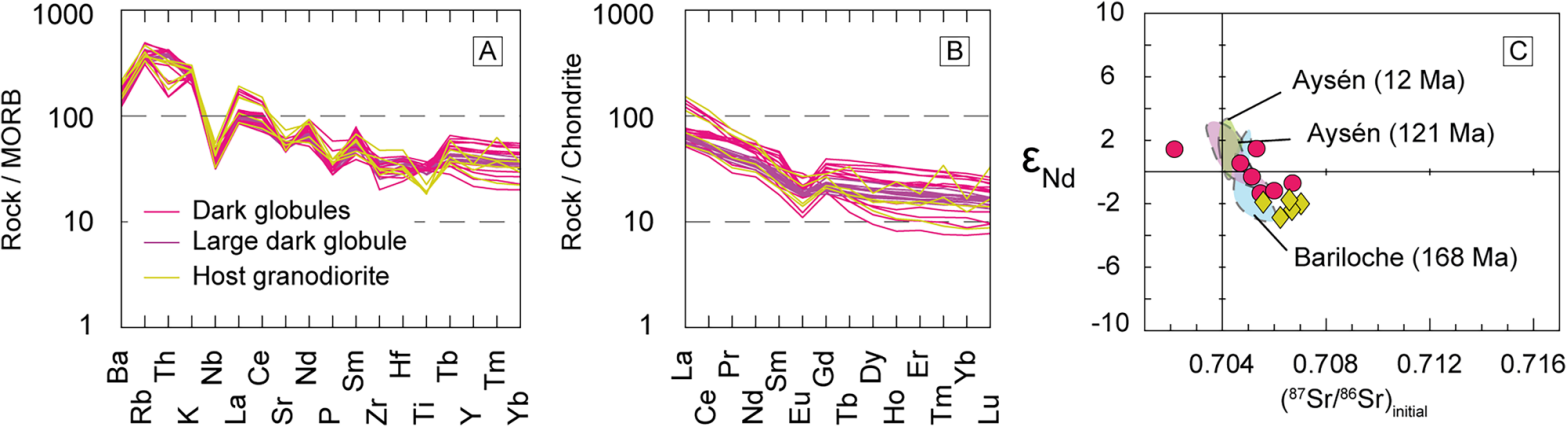
Trace element and isotopic diagrams from the Gerena samples. (**A**) Mid ocean ridge basalt-normalised trace element spider diagram^52^, and (**B**) chondrite-normalized diagram^58^; all groups showcase relatively similar patterns with typical arc signature. (**C**) ƐNd compared to initial ^87^Sr/^86^Sr calculated for 340 Ma. The Gerena rocks exhibit a similar tendency to those found in Aysén and Bariloche from the Patagonian batholith^31^. Granodiorites show a stronger crustal affinity than dark globules, suggesting assimilation of metapelitic host rocks.

Regarding trace elements, all analysed samples show relatively similar patterns, with typical arc signatures. Notably, dark globules exhibit roughly similar patterns to those from the host granodiorites (Fig. 5a,b). Whole rock Sr and Nd isotopes were determined in a selected range of representative samples from both the dark globules and host granodiorites (Fig. 5c). A majority of the examined samples exhibit a consistent pattern, with the host granodiorites displaying higher crustal affinity. Only one of them (A423-18) stands apart from the rest, showing a stronger mantle affinity. The trend followed by the Gerena samples parallels that seen in the North Patagonian batholith, exemplified by Aysén and Bariloche^31^, and further highlighting the similitudes between the Gerena MMZ and other Andean-type batholiths.

## Discussion

### Unfractionated magmatism in the Gerena magma mingling zone

Intermediate magmatism from the Gerena MMZ showcases a compositional range between larger and smaller dark globules. Samples from the central part of the large dark globule overlap the composition of the smaller dark globules, while the geochemical patterns displayed by the edges of the large dark globule are more similar to the granodiorite (Fig. 2f). Observed differences within the large dark globule are coherent with those predicted experimentally by in-situ crystallization at the sidewalls of magma channels^28^. These differences are due to fractional crystallization occurring in the edges caused by temperature contrast with the host, further physically enhanced by the more viscous mushes accumulating towards the edges. Given these considerations it can be argued that larger globules are suboptimal places to sample unfractionated rocks, due to the small variations at the rims of the globules after intrinsic in-situ crystallization.

Although fractionation is possible at the level of emplacement, as it was identified in the large dark globule, the composition of dark globules remains almost identical among varied shapes, pointing to negligible fractionation. Dark globules are comparable in textures and compositions to mafic microgranular enclaves (MMEs), a ubiquitous feature of granite batholiths in Andean-type settings^32^. Mafic microgranular enclaves have been interpreted as fragments of synplutonic magma injections or to represent the input of mafic magma from the mantle that triggered crustal melting and granite melt generation^33,34^. Nevertheless, dark globules display clear differences with larger dark globules that could represent magma injections found in Gerena MMZ, the latter characterised by angular edges and size of meters. Only a narrow range of variation is found in Sr and Nd isotopes, in which the granodiorite samples are richer in evolved components. This points to a possible crustal contaminant, enriched in radiogenic Sr and depleted in radiogenic Nd (Fig. 5c). The implication is that dark globules, on being cogenetic with the host granodiorites, were protected of contamination by their solid state and the surrounding felsic granodiorite during ascent and cooling. Thus, the evolved geochemical features such as the high silica content, low-MgO or isotopic values that characterise dark globules can be considered inherited from the source. However, it is noteworthy that trace element patterns (Fig. 5a,b) show clear similarities between dark globules and the comparatively more evolved granites. This observation is apparently contradictory when considering the differences in major elements between the two groups and can only be explained by the strong control imposed by accessory phases in granitic systems. Such issue is well-documented in granite petrogenesis, where processes like entrainment, fractionation or dissolution of common accessory phases such as zircon, monazite and apatite, can exert a strong influence on the resulting trace element signature^35,36^. These phenomena are rather unpredictable, hindering the use of trace element for petrogenetic interpretations in the Gerena MMZ. For this reason, we rely on the other evidence, namely the overlap between the Gerena dark globules, the parental experimental models and the compositional gap, and conclude that dark globules (or the equivalent MMEs) from MMZ are optimal places to probe for magmas with limited fractionation.

### Relationship with the Seville Sierra Norte batholith

Once the dark globules have been identified as undifferentiated magma, the next logical step is to test their genetic relationship with the granites and mafic rocks of the Seville Sierra Norte batholith. For this purpose, a binary difference test was conducted to assess the reproducibility of a parental composition from its respective differentiate and residue. In other words, this method conducts a regression model through binary differences to test the likelihood of two selected compositions, representing a fractionated rock and a differentiation residue, resulting from fractionating a parental composition. The regression accuracy is represented by R-squared values and embodies the likelihood of the three chosen compositions being related by differentiation (details on this method can be found in the Data Repository). Three representative dark globule and granite pairs were selected for geochemical modelling (points with thicker contours in Figs. 3, 4). These samples were intendedly collected in adjacent areas for this specific purpose. Regarding the residue, it is important to note that while the use of granite compositions for this purpose is relatively straightforward given their relative homogeneity due to their cotectic behaviour, the use of residue compositions is more challenging. This is mainly because the geochemistry of cumulate rocks, which likely result from multiple differentiation processes, is practically undistinguishable from that of differentiation residues. Additionally, olivine-rich cumulate rocks may be present in the Seville Sierra Norte batholith as a result of minor basaltic intrusions^27^. Since this model aims for a composition in equilibrium with a dioritic precursor represented by the dark globules (i.e., compositions resulting from a single differentiation process), selecting an accurate cumulate composition is a critical prerequisite. Taking these factors into account, we selected a composition that is coherent with the rest of the Seville Sierra Norte data, and close to the density maxima from the comparative series after the Guadalupe Igneous Complex in most of the diagrams. For comparison purposes, we also tested a reported cumulate from the literature^26^ (Figs. 3, 4, grey points with black contours).

**Figure 6:**
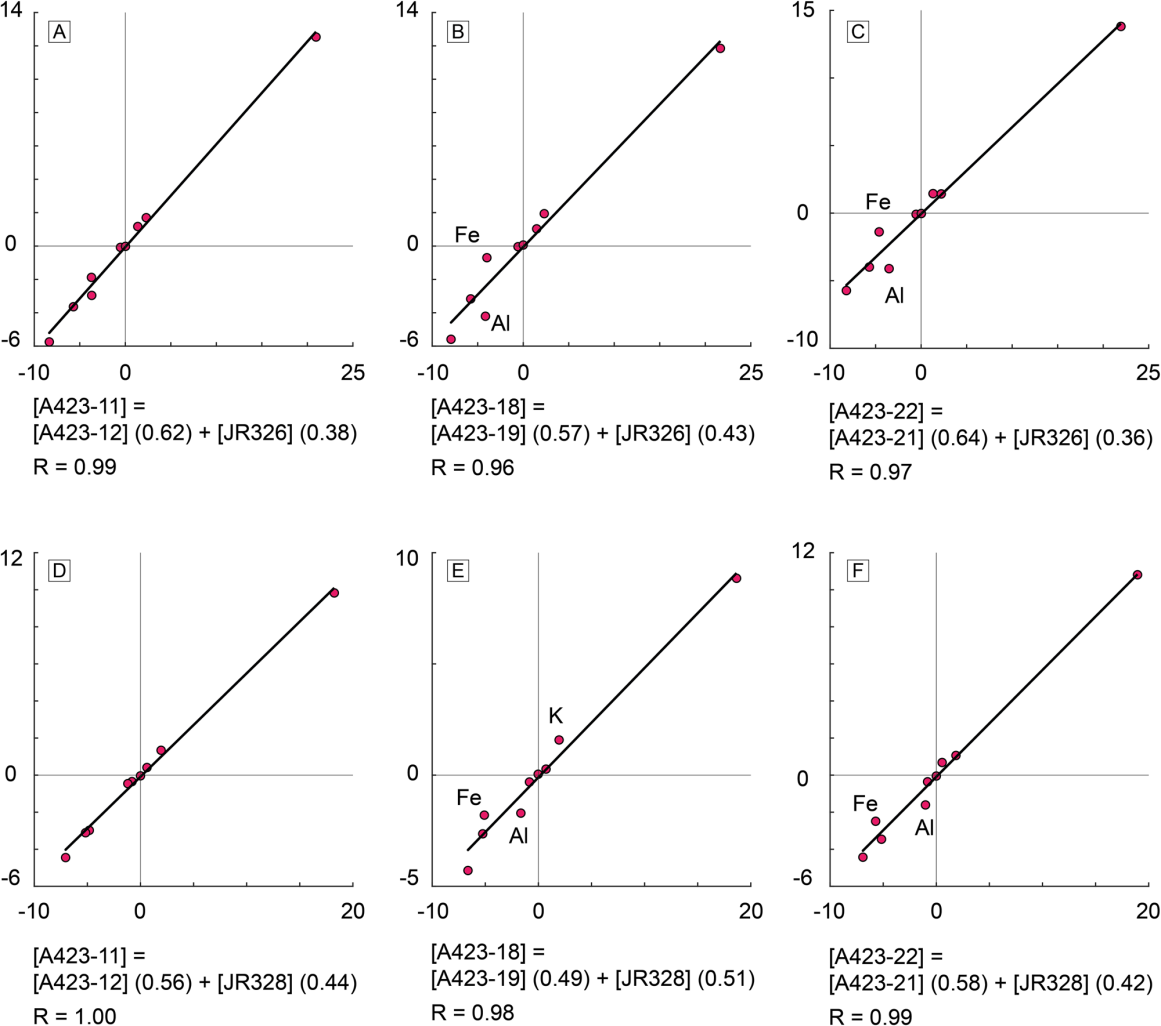
Binary difference test between the Gerena dark globules, differentiates and residual rocks from the Seville Sierra Norte batholith. Selected samples for each test are available in each diagram. Tests (**A–C**) are conducted with a cumulate composition, while tests (**D–F**) are conducted with a residual composition presumably with less cumulate affinity. All tests yield excellent correlations between the selected samples, implying that the dark globules can reproduce the fractionated and residual compositions through differentiation.

Results of the modelling are available in Fig. 6. Regardless of the used compositions, all tests yielded an excellent correlation with R-squared values of > 0.96. Tests using cumulate composition yields worse correlations for Fe and Al (Fig. 6a–c), likely due to accumulation of spinel. This can also be explained by small degrees of assimilation of host hornfels, supported by the presence of migmatization (Fig. 2f) and the slightly higher crustal affinity displayed by the granodiorites (Fig. 5c). Comparatively, a small improvement is observed in the selected residue composition (Fig. 6d–f). This observation is coherent with a parental melt of circa 60 wt% SiO_2_ resulting in a residue with basaltic-like composition (SiO_2_ ≈ 50 wt%). Moreover, variations among residual compositions having effect in the order of centesimals supports the strong genetic relation between the dark globules and the granodiorites. Although this test can be applied to trace element values, results may be unreliable for the reasons exposed above. Furthermore, while assimilation of small batches of host rocks may not have a significant effect in major elements (Figs. 3, 4), trace element and isotopes are a lot more sensible, with the latter showcasing slight deviations that are coherent with a contaminant (Fig. 4d).

### An intermediate precursor to Andean-type magmatism

Arc magmatism generation has been classically associated with basalt production from the peridotite mantle^37,38^, resulting in the felsic arc rocks by a number of processes, such as magma mixing and contamination^39^ or differentiation from a hydrous basaltic parental^40,41^. These models, however, entail important drawbacks that limit their viability, such as the elimination of an unseen ultramafic residue from the continents^38,42^, or their inability to account for the andesitic average composition of the continental crust^30^ without invoking complex multi-stage differentiation processes^37^. Not only that, but previous experimental work has also evidenced the inability to differentiate andesites from primary basalts, leading to inaccurate differentiate compositions^4^. Contrary to this, the use of an intermediate parental composition offers a solution to most of these challenges that, on top of it, is supported by existing experimental work (see references above). In this sense, dark globules from magma mingling zones can represent natural rocks matching the intermediate experimental models.

Even so, since a purely peridotitic mantle source is unable to produce intermediate magmatism^38,43^, a modification by either magma mixing or assimilation and fractional crystallization must be assumed^40^. Accordingly, pristine magmas of andesitic composition can be generated by melting of silicic diapirs coming from subducted mélanges of oceanic crust and sediments^44–46^ and their reaction with the peridotite mantle^47–50^. This consideration agrees with the data set showing the participation of evolved components even in the dark globules, a characteristic feature shared with diorites and granodiorites of various ages. Notably, similar patterns can be observed in Aysén and Bariloche in the North Patagonian batholith^31^, which were interpreted as resulting from melting of subducted mélanges incorporated by subduction and diapiric upwelling to hot zones of the suprasubduction mantle wedge. Consequently, major element geochemical trends of Andean-type batholiths follow cotectic lines that are reproduced experimentally by using a non-basaltic, dioritic (= andesitic) starting composition^4^. For those experiments, the composition of the parental diorite was modelled after the composition of the gap observed between granodiorites and diorites in the Patagonian batholith, following inference that large-scale fractionation is the cause of the gap^29,51^. Large-scale fractionation causes the parental magma to be the less represented by rocks, as these are mostly fractionated into liquids and residues. The same gap is observed in the whole Seville Sierra Norte batholith, in which dark globules represent the less abundant rocks of a general bimodal distribution (Fig. 3a,b), uncoincidentally plotting in the compositional gap between residues and differentiates. Note this is a common feature displayed by Andean-type magmatism, as shown by the comparative series (Fig. 3). Moreover, alternatives like magma mixing are unlikely due to the non-linear trends displayed by the Seville Sierra Norte batholith (Fig. 3). In contrast, differentiation is supported by the compositional correlation between the residues and differentiates and the dark globules as the potential parental magma (Fig. 6). These observations are further supported by the close match between the dark globules and the average bulk continental crust in most available diagrams (Figs. 3, 4), reinforcing the notion of continental growth in active margins through intermediate magmatism^49,52^.

A cogenetic relation between enclaves and host granodiorites (sensu lato) in Andean-type and post-collisional batholiths around the world is widely accepted^29,53,54^. The lack of reaction rims around MMEs, the compatibility of mineral assemblages between enclaves and host and the geochemical and isotopic similarities^55,56^ support a common magmatic origin. On top of that, most recent work on post-collisional MMEs show that enclaves may indeed represent the quenched parental magma of granite batholiths^56^. However, when probing for pristine parental magma in MMEs it is important to note that not all of them represent parental magmas. A study of mineral relations and whole chemical compositions of enclaves from the Tuolumne intrusive complex concluded that the most mafic MMEs represent cumulates that lost up to 50% liquid, yielding an estimated dioritic composition for the parental magmas of SiO_2_ = 60–62 wt%; CaO ≈ 5.2 wt%^32^. Such values are almost coincident with the model diorite composition (GAP model in Fig. 4) and the average values of the Gerena dark globules. Furthermore, the comparison with the diorites of the Kuna Crest lobe that represent the initiation of the Tuolumne batholith in Sierra Nevada (California) and, hence, the less fractionated pulse of magma^57^. In particular, the Zone I of the zoned lobe is the most primitive in terms of Sr–Nd isotopic ratios and has a major element composition that fairly matches that of the Gerena dark globules. Implication of these inferences is that mingling zones are a result of new pulses of parental magma intruding their own differentiates and residues.

## Concluding remarks

The Gerena magma mingling zone, located in the Sevilla Sierra Norte batholith, constitutes a world-class example featuring all typical characteristics of Andean-type magmatism. The host granodiorite is intruded by abundant intermediate magmas that appear in the form of dark globules of variable size. While larger dark globules hint to have undergone local fractionation, inference suggest that dark globules represent pristine magmas that have not undergone any significant differentiation. After modelling their correlation with differentiated rocks and cumulates from the Seville Sierra Norte batholith, further comparison with experimental and geochemical evidence suggests that these commonly occurring dark globules are, in turn, the parental to Andean-type batholiths. Altogether, we conclude that magma mingling zones and microgranular enclaves, both typical features of Andean-type batholiths, constitute optimal settings to probe for the unfractionated magmas, in which a pristine diorite (or andesite) composition can be identified as the parental magma of the system.

### Supplementary Information

Supplementary Information 1.

Supplementary Information 2.

## Data Availability

All data generated or analysed during this study are included in this published article, available in its Supplementary Files.
