# Supplementary material for: The pristine precursor of Andean-type magmatism preserved in magma mingling zones
Source: Sci Rep. 2024 Feb 29;14:5047. doi: 10.1038/s41598-024-55699-x (PMC10904382; doi:10.1038/s41598-024-55699-x)
Supplement: Supplementary file 2 — Supplementary Information 2. [file 41598_2024_55699_MOESM2_ESM.docx]

**Analytical techniques:**

Sampling of the Gerena magma mingling zone was made using classic techniques (hammer). Samples for the geochemical profile in the large dark globule were collected using core drill. All samples were collected in clean and fresh land cuts, devoid of any alteration.

Major elements were determined by XRF in the Instituto Andaluz de Ciencias de la Tierra (UGR-CSIC) and in the University of Oviedo, Spain (see table in data repository for details). The standard procedures are described in existing literature^1^. Analytical precision is better than 1% for major elements and better than ±5% for 100 ppm Zr. Trace elements, including REE were analysed on a AGILENT 7900 ICP-MS at the Center for Research in Sustainable Chemistry (CIQSO), University of Huelva, Spain, following digestion in a HF + HNO_3_ (8:3) solution, drying and re-dissolution in HNO_3_ and later HCl. The ICP-MS analyses were controlled by repeated analysis of the international rock standards AGV-1 and SARM, obtaining a precision better than 10%. Detection limits and analytical uncertainties for the analysed elements are less than 0.1 ppm.

0.1 g of the powder rock samples were attacked by a mixing of HF:HNO_3_:HCl (Merck® suprarpur quality) in Savillex® containers and on a hot plate at 90ºC^2^. After repeated etching, the samples were diluted to 2% in HNO_3_. The water used was of MilliQ quality. Trace elements were analysed by ICP-MS (Agilent7900) at the Center for Research in Sustainable Chemistry (CIQSO) of the University of Huelva. An external calibration (blank, 0.1 ppm, 1 ppm, 5 ppm, 10 ppm, 50 ppm, 100 ppm and 250 ppm) was used by a cocktail of multi-element standards:

- Multielement calibration standard-1: 10 μg mL-1 of Ce, Dy, Er, Eu, Gd, Ho, La, Lu, Nd, Pr, Sc, Sm, Tb, Th, Tm, Y, Yb in 5% HNO3.

- Multielement calibration standard-2A: 10 μg mL-1 of Ag, Al, As, Ba, Be, Ca, Cd, Co, Cr, Cs, Cu, Fe, Ga, K, Li, Mg, Mn, Na, Ni, Pb, Rb, Se, Sr, Tl, U, V, Zn in HNO3 5%.

- Multi-element calibration standard-4: 10 μg mL-1 of B, Ge, Mo, Nb, P, Re, S, Si, Si, Ta, Ti, W, Zr in HNO3 5%.

- Single element standard - Bismuth: 10 μg mL-1 of Bi in HNO_3_ 2%.

- Tin (Sn) standard: 10 μg mL-1 of Sn in HCl 5%.

- Antimony (Sb) standard: 10 μg mL-1 of Sb in HNO_3_ 1%.

- Hafnium (Hf) standard: 1000 μg mL-1 of HF in water with dilute HCl and traces of HF.

The possible drift during the perfomance of the analysis sequence was controlled with a multielemental solution of 10 ppb every 12 samples.

Supplementary table 4 shows the results of the Reference Standard Materials used (AGV-1: Andesite; SARM-4: North). In addition, SARM-4 was duplicated at the end of the sequence. For most elements of petrogenetic interest, the precision was better than 10%. The detection limit for most elements analysed is better than 0.1 ppm.

|  | ppm | ppm | ppm |  | ppm | ppm |
| --- | --- | --- | --- | --- | --- | --- |
|  | analysed | analysed duplicated | certificated |  | analysed | certificated |
| Reference | SARM-4 | SARM-4 (BIS) | SARM4 |  | AVG-1 | AGV-1 |
| Li | 4,5 | 4,6 |  |  | 9,8 | 12 |
| Be | 0,1 |  | 0,35 |  | 1,8 | 2,1 |
| Sc | 40,3 | 37,0 | 37,4 |  | 14,5 | 12 |
| V | 241 | 200 | 220 |  | 130 |  |
| Cr | 27,5 | 23,5 | 30 |  | 16,1 | 10 |
| Co | 58,3 | 52,9 | 58 |  | 15,5 | 15 |
| Ni | 117 | 103 | 120 |  | 17,7 |  |
| Cu | 9,6 | 9,2 | 14 |  | 48,8 | 60 |
| Zn | 91,4 | 77,5 | 68 |  | 132 |  |
| Ga | 14,5 | 13,3 | 16 |  | 24,5 | 20 |
| Ge | 0,4 | 0,3 |  |  | 3,0 | 1,25 |
| As | 0,1 | 0,1 | 0,4 |  | 2,3 |  |
| Se | 0,6 | 0,6 |  |  | 2,6 |  |
| Rb | 3,5 | 3,0 | 5 |  | 69,4 | 67 |
| Sr | 273 | 213 | 260 |  | 698 | 660 |
| Y | 5,7 | 5,2 | 7 |  | 18,5 |  |
| Zr | 11,7 | 10,1 | 14 |  | 243,1 |  |
| Nb | 1,6 | 0,2 |  |  | 14,3 |  |
| Mo | 0,9 | 0,4 |  |  | 1,7 | 2,7 |
| Cd | nd | nd | 0,07 |  | 0,2 |  |
| Sn | nd | nd |  |  | 1,6 |  |
| Sb | 0,3 | nd |  |  | 0,4 | 4,3 |
| Cs | nd | nd | 0,22 |  | 0,6 |  |
| Ba | 87,1 | 62,6 | 102 |  | 1235 | 1230 |
| La | 2,6 | 2,3 | 3 |  | 38,2 | 38 |
| Ce | 5,6 | 5,0 | 6 |  | 70,6 | 67 |
| Pr | 0,4 | 0,3 | 0,77 |  | 7,6 |  |
| Nd | 2,8 | 2,6 | 3,2 |  | 29,7 | 33 |
| Sm | 0,5 | 0,4 | 0,8 |  | 5,1 | 5,9 |
| Eu | 0,2 | 0,2 | 0,63 |  | 1,3 | 1,6 |
| Gd | 1,0 | 1,0 | 0,91 |  | 5,6 | 5 |
| Tb | 0,2 | 0,2 | 0,17 |  | 0,7 | 0,7 |
| Dy | 1,1 | 1,0 | 1 |  | 3,5 | 3,6 |
| Ho | 0,2 | 0,2 | 0,22 |  | 0,7 |  |
| Er | 0,7 | 0,6 | 0,67 |  | 1,9 |  |
| Tm | 0,1 | 0,1 | 0,1 |  | 0,3 |  |
| Yb | 0,7 | 0,6 | 0,7 |  | 1,6 | 1,72 |
| Lu | 0,1 | 0,1 | 0,2 |  | 0,3 | 0,27 |
| Hf | 0,5 | 0,3 |  |  | 4,7 | 5,1 |
| Ta | 2,6 | 0,7 | 0,07 |  | 1,4 | 0,9 |
| W | 1,0 | 0,3 | 0,19 |  | 0,9 |  |
| Tl | 0,3 | 0,1 |  |  | 0,5 |  |
| Pb | 2,2 | 2,0 | 2,48 |  | 12,0 | 36 |
| Bi | 0,1 | 0,1 |  |  | 0,1 |  |
| Th | 0,4 | 0,3 | 0,42 |  | 5,9 | 6,5 |
| U | 0,3 | 0,3 | 0,28 |  | 1,8 | 1,92 |

*Supplementary table 4. Results of the analyses of the Reference Standard Materials used.*

For Sr and Nd isotope determinations, representative samples from the Gerena samples were digested in a clean room using ultraclean reagents. Samples were selected with the intention to cover all compositional range and check for isotopic equilibrium. They were then analysed by thermal ionization mass spectrometry (TIMS) in a Finnigan Mat 262 spectrometer (Universidad Complutense de Madrid) after chromatographic separation with ion-exchange resins. Normalization values were ^86^Sr/^88^Sr = 0.1194 and ^146^Nd/^144^Nd = 0.7219.

**Geochemical modelling:**

Geochemical modelling was based on a binary difference test. The binary difference test assesses the reproducibility of a hybrid composition by numerically mixing two components using a regression model. The calculation begins with a mass balance calculation:

$$H=A\left( x \right)+B\left( 1-x \right)$$

Where H is the hybrid, A and B are the two components mixed to reproduce the hybrid composition, and x is the mixing proportion. In this case, the hybrid (composition H) represents a parental composition that undergoes fractionation and produce a differentiate and a residue (compositions A and B). Clearing x in this expression results in

$$H-B=x\left( A-B \right)$$

This relation resembles the equation of a line in which x is the only unknown. Components H, B and A are the major elements composition of the used samples, and the equation is solved for each element. Coordinates H – B and A – B are then used to fit a regression model, yielding a numerical approximation for x. The resulting r-squared value from this regression represents the likelihood of components A and B resulting from differentiation of the hybrid/parental composition H.

**References:**

^1^Baedecker, P.A., Methods for geological analysis. US Geological Survey Bulletin. U.S Geological Survey Bulletin 1770 (1987).

^2^De la Rosa, J. D., Chacón, H.; Sánchez de la Campa, A.; Carrasco, R. & Nieto, J.M. Metodología y análisis de elementos trazas-REE mediante ICP-MS del standard SARM 1 granito y SARM 4 norita. III Congreso Ibérico de Geoquímica, Zaragoza, España. Actas: 435-438 (2001).
